# An eight-long non-coding RNA signature as a candidate prognostic biomarker for bladder cancer

**DOI:** 10.18632/aging.102225

**Published:** 2019-09-03

**Authors:** Penghu Lian, Qian Wang, Ya Zhao, Cheng Chen, Xiacheng Sun, Hanzhong Li, Jianhua Deng, Hongmei Zhang, Zhigang Ji, Xuebin Zhang, Qichao Huang

**Affiliations:** 1 Department of Urology, Peking Union Medical College Hospital, Chinese Academy of Medical Sciences and Peking Union Medical College (CAMS & PUMC), Beijing 100730, China; 2 Department of General Surgery, Tangdu Hospital, Fourth Military Medical University, Xi’an 710032, China; 3 Biomedicine Application Laboratory, School of Life Science and Technology, Xidian University, Xi’an 710038, China; 4 State Key Laboratory of Cancer Biology and Experimental Teaching Center of Basic Medicine, Fourth Military Medical University, Xi’an 710032, China; 5 Department of Clinical Oncology, Xijing Hospital, Fourth Military Medical University, Xi’an 710032, China

**Keywords:** bladder cancer, long non-coding RNA, recurrence free survival, prognosis

## Abstract

Backgroud: Bladder cancer (BLCA) is one of the most fatal types of cancer worldwide. However, there are limited methods for us to provide a prognostic prediction of BLCA patients. Therefore, we aimed at developing a lncRNA signature to improve the prognosis prediction of BLCA.

Results: An eight-lncRNA signature was significantly associated with recurrence free survival in BLCA patients from both discovery and validation groups. Furthermore, genes involved in the signature were enriched in extracellular matrix organization pathway. Finally, functional experiments demonstrated that six out of the eight lncRNAs significantly regulated the invasion of BLCA cells.

Method: A total of 343 BLCA patients from The Cancer Genome Atlas (TCGA) were employed and randomly divided into training (n=172) and validating (n=171) groups. The lncRNA expression profiles of BLCA patients were screened and a risk-score formula were created and validated according to the Cox regression analysis. Next, WGCNA method was employed to cluster genes that highly correlated with the risk scores based on the profiling data of TCGA dataset and transwell assay was also performed to further investigate the role of these lncRNAs.

Conclusions: Our results suggested that the eight-lncRNA signature was a candidate prognostic biomarker for predicting tumor recurrence of patients with BLCA.

## INTRODUCTION

Bladder cancer (BLCA) is the ninth most common cancer worldwide, accounting for 7% of all cancers and 3% of all cancer deaths [1]. Despite diverse treatment methods including surgery, radiotherapy, chemotherapy, and Bacillus Calmette-Guérin (BCG) therapy [2], the risk of recurrence after 5 years ranges from 50% to 90% in non-muscle-invasive bladder cancer [3]. The high recurrence rate of bladder cancer is partly due to the lack of effective prognostic biomarkers. Therefore, developing an effective screening method for early detection of bladder cancer is critical.

Long non-coding RNAs (lncRNAs) are defined as RNA transcripts longer than 200 bases that that are not translated into proteins [4, 5]. Although the functions of only a limited number of lncRNAs have been fully explained, numerous studies have suggested that lncRNAs are involved in many biological processes, including cell proliferation [6], differentiation [7], and chromatin modification [8]. Accumulating evidence has suggested that lncRNAs are frequently deregulated in cancer cells and involved in the development and progression of cancers. For example, in prostate cancer, lncRNA HULC was up-regulated in cancer tissues and associated with a poor overall survival of prostate cancer patients [9]. Li et al. have found that lncRNA FAL1 was positive in hepatocellular carcinoma (HCC) tissues and functioned as an oncogene [10]. Ye et al. have reported that LINC00460 might be a potential prognostic biomarker in lung cancer [11]. Recent studies have also demonstrated emerging roles of lncRNAs in BLCA. For instance, Zhu et al. have found that lncRNA LSINCT5 was significantly over-expressed in human BLCA specimens, and facilitated BLCA progression by enhancing Wnt/β-catenin signaling activation and epithelial mesenchymal transition (EMT) [12]. These findings strongly suggested lncRNAs could serve as diagnostic and prognostic biomarkers in human cancer.

Currently, with the advancements in transcriptome profiling, lncRNA profiling could be achieved by mining previously published gene expression microarray data. Therefore, searching a lncRNA signature might be better strategy to find a novel biomarker for the accurate prognosis prediction of patients with cancer. For example, Yang et al. have identified a six-lncRNA signature associated with recurrence of ovarian cancer [13]. In addition, Song et al. developed a lncRNA signature with prognostic value for survival outcomes of gastric cancer [14]. Subsequent studies also discovered lncRNA signatures that were significantly associated with the survival of patients with thyroid papillary carcinoma [15], pancreatic cancer [16], and oesophageal squamous cell carcinoma [17]. However, the prognostic power of lncRNA signatures in predicting the survival of patients with BLCA has not yet been investigated.

In the present study, we conducted a comprehensive study of lncRNA expression profiles in a cohort of 343 BLCA patients from The Cancer Genome Atlas (TCGA) database. We identified an eight-lncRNA signature with the ability to predict the recurrence free survival of patients with BLCA and validated their biological function in human BLCA cells.

## RESULTS

### Derivation of an eight-lncRNA prognostic signature from BLCA patients in the training dataset

The BLCA samples (n=343) were randomly divided into training and validating series (Table 1). There is no significant difference in age, race, pathological grade, disease stage and recurrence status between the two series except the proportion of male patients (68% VS 81.3%). To explore the correlation between lncRNA expression signatures and the recurrence free survival (RFS) of BLCA patients, we firstly screened the lncRNA expression profile from training series (n= 172) and then evaluated in the validating series (n=171). By subjecting the lncRNA expression data derived from the training series to univariable Cox proportional hazards regression analysis, we identified some lncRNAs that were strongly correlated with patients’ recurrence free survival (*P* value were less than 0.05). As a result, 8 genes were finally screened out as the predictors. Among these genes, positive coefficients indicated that the higher expression levels of six genes (APCDD1L-AS1, FAM225B, LINC00626, LINC00958, LOC100996694 and LOC441601) were associated with shorter survival. The negative coefficients for the remaining two genes (LOC101928111 and ZSWIM8-AS1) indicated that their higher levels of expression were associated with longer survival (Table 2).

**Table 1:** Clinical features of BLCA patients in the training and validating groups.

**Features**	**Training group (n=172)**	**Validating group (n=171)**	***P* value**
Age (years), no (%)			
≤70	101 (58.7)	98 (57.3)	0.791
>70	71 (41.3)	73 (42.7)	
Gender, no (%)			
Male	117 (68.0)	139 (81.3)	0.005
Female	55 (32.0)	32 (18.7)	
Race, no (%)			
Asian	15 (8.7)	24 (14.0)	0.424
African American	9 (5.2)	11 (6.4)	
Caucasian	141 (82.0)	130 (76.0)	
Others	7 (4.1)	6 (3.5)	
Pathological grade, no (%)			
Low	9 (5.2)	11 (6.4)	0.635
High	163 (94.8)	160 (93.6)	
Disease stage, no (%)			
I+II	60 (34.9)	57 (33.3)	0.876
III	64 (37.2)	62 (36.3)	
IV	48 (27.9)	52 (30.4)	
Recurrence status (%)			
Yes	71 (41.3)	70 (40.9)	0.948
No	101 (58.7)	101 (59.1)	

Abbreviations: BLCA: bladder cancer.

**Table 2:** Eight lncRNAs significantly associated with the RFS of BLCA patients in the training group.

**Gene symbol**	**RefSeq Transcript ID**	**Ensembl**	**HR^a^**	**95%CI of HR**	**Coefficient^a^**	***P*-value^a, b^**
APCDD1L-AS1	NR_034147	ENSG00000231290	1.45	1.13-1.86	0.37	0.003
FAM225B	NR_024376	ENSG00000231528	8.41	2.22-31.80	2.13	0.002
LINC00626	NR_024160	ENSG00000225826	1.98	1.17-3.34	0.68	0.011
LINC00958	NR_038904	ENSG00000251381	1.03	1.00-1.05	0.03	0.030
LOC100996694	NR_121639	ENSG00000250392	1.20	1.04-1.38	0.18	0.015
LOC101928111	XR_251299	ENSG00000222020	0.57	0.36-0.91	-0.56	0.019
LOC441601	NR_003034	ENSG00000255042	1.11	1.04-1.19	0.11	0.003
ZSWIM8-AS1	XR_945852	ENSG00000272589	0.01	0.00-0.99	-4.26	0.049

Abbreviations: *HR*: Hazard ratio; BLCA: bladder cancer; RFS: recurrence free survival.

^**a**^ Derived from the univariable Cox proportional hazards regression analysis in the 172 test series patients

^**b**^ Obtained from permutation test repeated 10,000 times

### An eight-lncRNA signature predicts survival of BLCA patients in the training dataset

**Figure 1:**
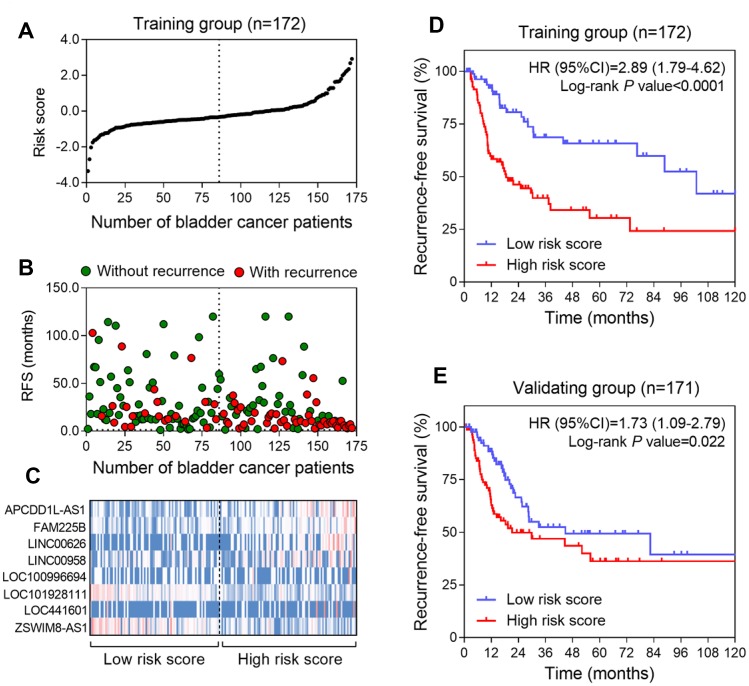
**Construction of the eight-lncRNA risk model of BLCA.** (**A**) lncRNA signature risk score distribution in the training group. (**B**) BLCA patients’ survival status in the training group. (**C**) Heatmap of the lncRNA expression profiles. Rows represent lncRNAs, and columns represent patients. The black dotted line represents the median lncRNA risk score cutoff dividing patients into low-risk and high-risk groups. Red: high expression; Blue: low expression. (**D** and **E**) Kaplan-Meier analysis for the recurrence free survival of BLCA patients in training series (**D**) and in validating series (**E**).

To investigate whether the eight-lncRNA signature could provide an accurate prediction of RFS in BLCA patients, a risk-score formula was created according to the expression of these 8 lncRNAs for RFS prediction, as follows: Risk score = (0.37 × expression value of APCDD1L-AS1) + (2.13 × expression value of FAM225B) + (0.68 × expression value of LINC00626) + (0.03 × expression value of LINC00958) + (0.18 × expression value of LOC100996694) + (−0.56 × expression value of LOC101928111) + (0.11 × expression value of LOC441601) + (−4.26 × expression value of ZSWIM8-AS1). Then the eight-lncRNA signature risk score were calculated for each patient in the training series. As such, patients were ranked according to their risk scores and divided into a high-risk group (n = 86) or a low-risk group (n = 86) using the median risk score of the training series as the cutoff point (Figure 1A). As expected, a higher recurrence rate was noted for BLCA patients with high-risk scores than for those with low-risk scores (Figure 1B). Moreover, tumor tissues obtained from patients with high-risk scores tended to express high level of risky lncRNAs (APCDD1L-AS1, FAM225B, LINC00626, LINC00958, LOC100996694 and LOC441601) in their tumors, whereas tumor tissues from patients with low-risk scores tended to express high level of protective lncRNAs (LOC101928111 and ZSWIM8-AS1) (Figure 1C). Kaplan–Meier curves showed that, in the training series (n = 172), patients in the high-risk group had a significantly shorter RFS than those in the low-risk group (HR=2.89, 95%CI =1.79-4.61, log-rank test *P*<0.0001) (Figure 1D). In detail, RFS rates of patients in the high-risk group were 48.6% at 24 months, 33.1% at 48 months, 27.8% at 72 months and 25.3% at 96 months, versus 83.1%, 70.3%, 65.7% and 60.2% in the low-risk group, respectively.

### Validation of the eight-lncRNA signature for survival prediction

To confirm our findings, the prognostic power of the eight-lncRNA signature was further evaluated in the validating series. According to the same risk formula, patients in this cohort were divided into high-risk group (n = 86) and low-risk group (n = 85). Kaplan-Meier curves revealed that the high-risk scores of eight-lncRNA signature were significantly associated with lower RFS of BLCA patients (HR = 1.73, 95% CI: 1.09-2.79, p =0.022) (Figure 1E), which were similar to those observed in the training series.

### Survival prediction by the eight-lncRNA signature is independent of clinical features

We performed multivariable Cox regression analysis to evaluate whether the eight-lncRNA signature was an independent predictor of BLCA patient’s survival. Clinical features including age, gender, race, pathological grade and TNM stage were defined as covariates. Our results from the validation series showed that the prognostic power of the eight-lncRNA signature risk score (high-risk group vs. low-risk group, HR = 1.73, 95% CI: 1.09-2.79, *P* = 0.022) was independent of these clinical features (Table 3). In addition, similar results were also obtained from all the BLCA samples (n=343, Table 4). Moreover, we performed stratified analysis to identify the subgroups of appropriate using the eight-lncRNA signature. The results showed effective prognostic power of the eight-lncRNA signature in female patients from the validation series (HR = 5.58, 95% CI: 1.88-20.12, *P* = 0.003; HR = 3.42, 95% CI: 1.78-6.58, *P* < 0.001). The results also showed effective prognostic power of the eight-lncRNA signature in Caucasians patientsand high pathological grade patients.

**Table 3:** Multivariable Cox regression analysis on the association between eight-lncRNA signature and RFS of BLCA patients in validation series.

**Variables**	**Total number**	**High risk score**			**Low risk score**		**HR (95%CI)**	**P value**
		**Case number**	**MST (month)**		**Case number**	**MST (month)**		
Overall	171	86	21.2		85	44.8	1.73 (1.09-2.79)	0.022
Age (years)								
≤ 70	98	53	18.3		45	25.6	1.57 (0.87-2.84)	0.135
> 70	73	33	29.9		40	82.4	1.85 (0.86-4.07)	0.116
Gender								
Male	139	71	29.9		68	28.7	1.35 (0.81-2.27)	0.248
Female	32	15	12.0		17	NA	5.58 (1.88-20.12)	0.003
Race								
Caucasian	130	69	29.9		61	82.4	1.81 (1.06-3.07)	0.029
Others	41	17	19.4		24	22.6	1.56 (0.57-4.67)	0.378
Pathological grade								
High	160	82	21.2		78	44.8	1.71 (1.07-2.77)	0.025
TNM stage								
I+II	57	27	NA		30	NA	0.89 (0.32-2.45)	0.814
III	62	34	21.2		28	NA	2.84 (1.16-6.41)	0.023
IV	52	25	11.4		27	25.3	1.97 (1.04-4.22)	0.041

Abbreviations: MST: median survival time; *HR*: Hazard ratio; BLCA: bladder cancer; RFS: recurrence free survival.

**Table 4:** Multivariable Cox regression analysis on the association between eight-lncRNA signature and RFS of all BLCA patients.

**Variables**	**Discovery group**		**Validation group**		**Combination**	
	**HR (95%CI)**	**P value**	**HR (95%CI)**	**P value**	**HR (95%CI)**	**P value**
Overall	2.89 (1.80-4.63)	<0.001	1.73 (1.09-2.79)	0.022	2.23 (1.60-3.11)	<0.001
Age (years)						
≤ 70	3.00 (1.65-5.45)	<0.001	1.57 (0.87-2.84)	0.135	2.17 (1.43-3.31)	<0.001
> 70	2.72 (1.25-5.91)	0.012	1.85 (0.86-4.07)	0.116	2.26 (1.31-3.92)	0.003
Gender						
Male	3.02 (1.67-5.48)	<0.001	1.35 (0.81-2.27)	0.248	1.89 (1.28-2.80)	0.001
Female	2.62 (1.20-5.71)	0.012	5.58 (1.88-20.12)	0.003	3.42 (1.78-6.58)	<0.001
Race						
Caucasian	2.58 (1.56-4.27)	<0.001	1.81 (1.06-3.07)	0.029	2.18 (1.52-3.14)	<0.001
Others	6.43 (1.64-25.24)	<0.001	1.56 (0.57-4.67)	0.378	2.40 (1.05-5.51)	0.025
Pathological grade						
High	2.89 (1.80-4.65)	<0.001	1.71 (1.07-2.77)	0.025	2.21 (1.58-3.09)	<0.001
TNM stage						
I+II	1.27 (0.44-3.69)	0.633	0.89 (0.32-2.45)	0.814	1.12 (0.54-2.30)	0.758
III	3.75 (1.67-8.38)	0.002	2.84 (1.16-6.41)	0.023	3.30 (1.84-5.94)	<0.001
IV	3.11 (1.54-6.30)	0.005	1.97 (1.04-4.22)	0.041	2.30 (1.41-3.75)	<0.001

### Identification of eight lncRNA signature associated biological pathways and processes

**Figure 2:**
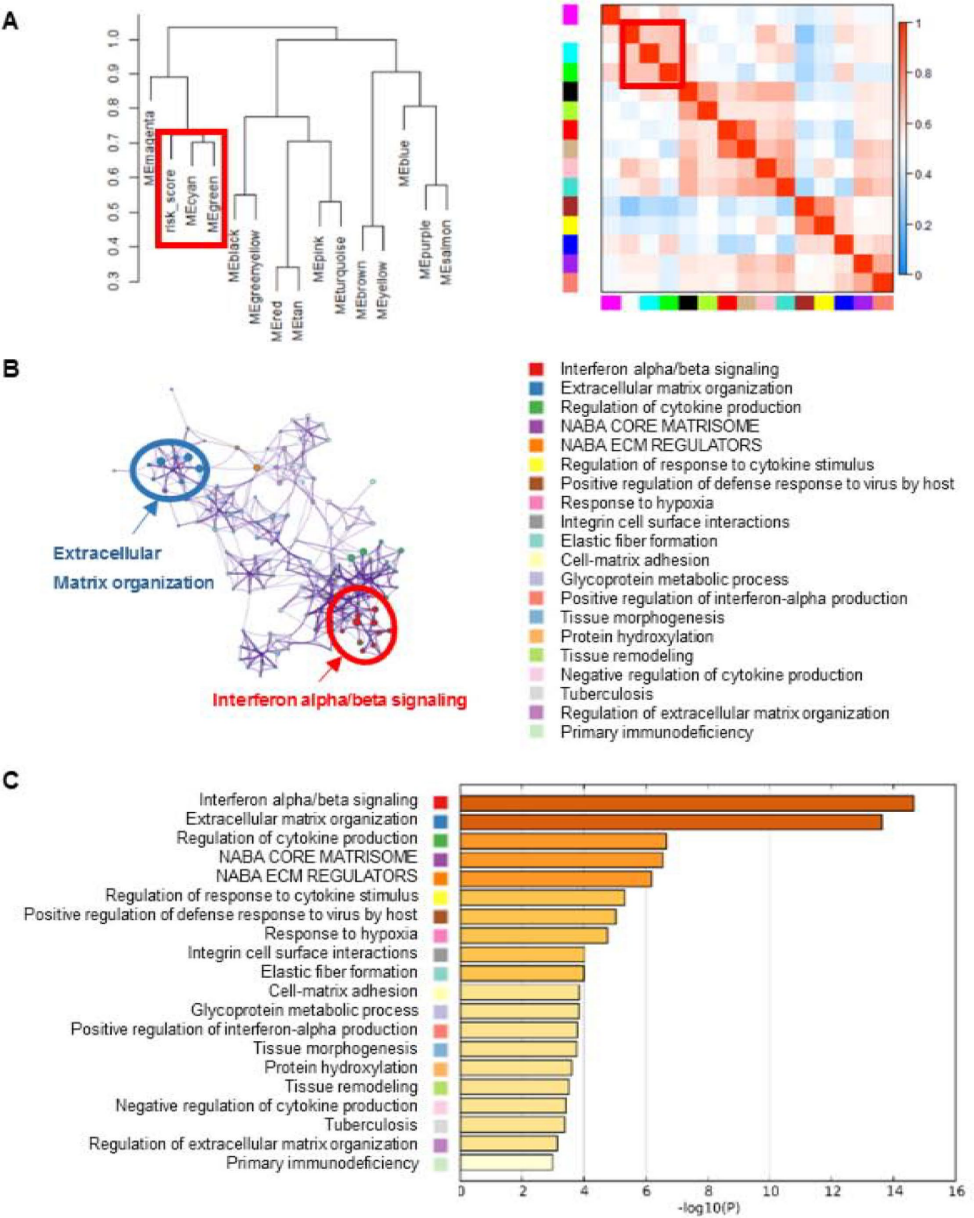
**Gene enrichment analysis of the lncRNA-signature.** (**A**) WGCNA method were performed to cluster genes that highly correlated with the risk scores. Clustering dendrogram and eigengene adjacency heatmap were generated using genes associated with the eight-lncRNA signature. (**B**) The pathways related with eight-lncRNA signature were clustered using Metascape. The cluster was made up of the best enriched pathways. The top 20 enriched pathways were shown (right panel) and the top 2 enriched pathways were marked (left panel). (**C**) The histogram of the top 20 enriched pathways associated with risk score was arranged by -Log_10_P value. Each bar represented one enriched term and was colored by -Log_10_P value.

In order to explore the potential mechanisms of the eight lncRNA signature, we performed WGCNA method to cluster genes that highly correlated with the risk scores based on the profiling data of TCGA dataset. We identified a total of 14 modules and found that cyan and green modules were most significantly correlated with the risk-score (Figure 2A). Pathway enrichment analysis was then performed using the genes in cyan and green modules. As shown in Figure 2B and 2C, genes were significantly enriched in cancer-related networks, including extracellular matrix organization pathways, interferon alpha/beta signaling, cytokine production pathway et al., suggesting the activation of these pathways might contribute to higher mortality risk in patients with high risk scores.

### The eight lncRNA signature regulates the invasion ability of BLCA cell lines

**Figure 3:**
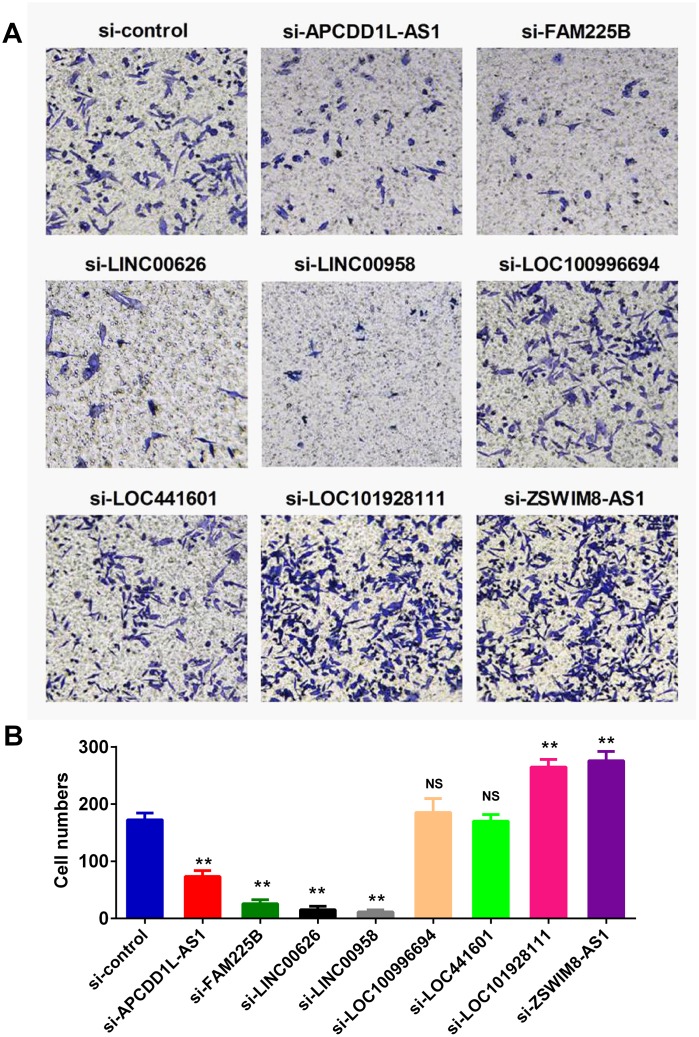
**lncRNAs regulated the invasion ability of BLCA cell lines.** Invasion assay was employed to monitor the effect of lncRNA expression on cell invasiveness. Specific small interfering RNAs (siRNAs) were used to knockdown the lncRNAs expression in human BLCA BIU-87 cells. Representative images of invasion assay were presented in (**A**), and the qualification result was shown in (**B**).

Defects in extracellular matrix organization have long been considered a hallmark of a transformed cellular phenotype and may promote tumor metastasis and progression. We therefore evaluated the effects of the eight lncRNAs on cell invasion by small interfering RNAs (siRNAs) in human BLCA cell line-BIU-87. Our data showed that knockdown of CDD1L-AS1, FAM225B, LINC00626 or LINC00958 significantly inhibited cell invasion. In contrast, knockdown of LOC101928111 or ZSWIM8-AS1 exerted opposite effects. It is noteworthy that the effects of LOC100996694 and LOC441601 on cell invasion were not obvious (Figure 3).

## DISCUSSION

Accumulating evidence suggested that lncRNA are involved in cancer development and these dysregulated lncRNAs have already shown great potential as novel molecular biomarkers in early diagnosis, therapeutic process monitoring and prognostic evaluation of cancer [4]. Nevertheless, single lncRNA may not be accurate enough for predicting the prognosis of cancer patients [18]. In recent years, transcriptional profiling analyses have discovered a number of tissue-specific lncRNAs in normal tissues and dysregulated lncRNAs in a variety of human cancers. Therefore, the expression profile-based prognostic lncRNA signature has been investigated in breast cancer, colorectal cancer, gastric cancer and lung cancer [4, 19–21]. However, the prognostic values of lncRNA signature in BLCA have not yet been investigated. To explore the prognostic lncRNA signature, we profiled lncRNA by mining the existing lncRNA expression data in TCGA and identified an eight-lncRNA signature which was closely related to the prognosis of patients with BLCA.

Although men are more likely to suffer from bladder cancer, women present generally with more advanced disease and have worse oncologic outcomes [22]. One possible reason lies in the difference in sex steroid hormones and their receptors between men and women, which plays an important role in bladder cancer development and progression. Interestingly, in the present study, we find that the prognostic values of the eight-lncRNA signature were more favorable in female. In addition, the relationship between race and BLCA is also complex. DeDeugd et al. found that African-Americans initially present with more aggressive BLCA, however, African-Americans actually have an improved overall survival compared with Caucasians [23]. However, Schinkel et al. reported that white and black patients with BLCA were not significantly different in overall and recurrence-free survival regardless of muscle invasion [24]. In the present study, when the patients with BLCA were stratified by race, the results showed that for the Caucasians, they can be divided into either a high-risk group with shorter survival or a low-risk group with longer survival according to the eight-lncRNA signature. For other races, however, the model has lost its prognostic power.

Accumulating evidence documented that non-protein coding genes play important roles in cancer development and progression. For example, Seitz et al. have demonstrated that LINC00958 was upregulated in bladder cancer tissues. While, knock-down of LINC00958 inhibited the invasion and migration of bladder cancer cells [25]. Moreover, Yazarlou et al. have found that the expression of LINC00958 in urinary exosomes are potential diagnostic bio-markers in bladder cancer [26]. In addition, Guo et al. have reported that LINC00958 could accelerate gliomagenesis through regulating miR-203/CDK2 axis [27]. In accordance with these studies, our data also showed that knockdown of LINC00958 significantly inhibited invasion of BLCA cells. However, the biological functions of the other seven signature lncRNAs were not reported before. Thus, our results still need to be further investigated and validated in human cancers.

Several limitations of this study should be noted. First, the small sample size and lack of validation data from an independent cohort. Second, there are two lncRNAs involved in the signature (LOC100996694 and LOC441601) had no effects on cell invasion without a reasonable explain. Finally, the detailed mechanism still needs further experiments.

In summary, we identified an eight-lncRNA signature, which was significantly associated with recurrence free survival in BLCA patients. Further analysis revealed that genes involved in the signature were enriched in extracellular matrix organization pathway. Moreover, six out of the eight lncRNAs significantly regulated the invasion capability of BLCA cells.

## MATERIALS AND METHODS

### Human BLCA cell line

The BIU-87 human BLCA cell line was preserved in our department and routinely cultured in DMEM medium supplemented with 10% FBS. All siRNAs were designed and synthesized by GenePharma (Shanghai, China). The siRNAs were transfected with LipoGene™ 2000 PLus Transfection Reagent (US Everbright Inc. Suzhou, China) reagent according to the manufacturer’s protocol.

### Bladder cancer datasets and patient information

Clinical information and the FPKM (Fragments Per Kilobase of transcript per Million fragments mapped) values for LncRNAs in BLCA tissues were directly download from TCGA using an online software (https://shengxin.ren). Then the clinical information files were converted into matrix format and the ENSG ID in RNA-Seq were also converted into gene Symbol using the same software. To analyze the correlation of lncRNA expression signatures with the recurrence free survival of bladder cancer patients, a total of 343 patients with recurrence information were enrolled in the study, after filtering out samples without clinical survival information. Then, the 343 patients were divided into training and validating groups randomly according to their batch numbers.

### Data processing and risk-score calculation

The lncRNAs were subjected to univariable Cox regression proportional hazards regression analysis to select lncRNAs which were associated with RFS of BLCA patients. Those lncRNAs with a statistical significance in univariable Cox regression were then selected into multivariable Cox regression to obtain the coefficients. By linearly combining the expression value of selected lncRNAs weighted by their coefficients, a risk-score formula was constructed as following: $Risk\;Score\;(RS)=\sum_{i=1}^{N}\left(Expi*Coei\right)$. *N* is the number of prognostic lncRNA genes; *Expi* is the expression value of lncRNA, and *Coei* is the estimated regression coefficient of lncRNA in the multivariable Cox regression analysis. Such a risk score model is fully taken into account in the power of each of the prognostic lncRNA genes. Each patient has been given a risk score that is a linear combination of the expression levels of the significant lncRNAs weighted by their respective Cox regression coefficients.

### Weighted correlation network analysis (WGCNA)

Considering that our risk score model was based on the expression levels of eight lncRNA, we construct a “risk scrore-gene” co-expression analysis to predict the potential biological function of our model. In this study, we select WGCNA method to find the gene modules associated with our risk scores using the R package “WGCNA” according to previous reports [28]. The soft thresholding power was selected to 9 to produce a weighted network. The enrolled genes were hierarchically clustered into 14 modules.

### Pathway enrichment analysis

The cyan and green modules, the most significant modules being associated with risk score, were picked out to perform the pathway enrichment analysis. Pathway enrichment was carried out using an online-based web tool “Metascape” (http://metascape.org/). The significance threshold of false discovery rate (FDR) for the significantly enriched biological processes and pathways was set at 0.05.

### Invasion assay

The invasive capability of BLCA cells was determined by the transwell assay. The membrane was coated with the Matrigel (200 ng/mL; BD Biosciences). Then BLCA cells transfected with lncRNA siRNAs or control siRNA were seeded in the upper chamber. The DMEM medium supplemented with serum was placed in the lower chamber. The cells on the lower side of the filters were defined as invasive cells.

### Statistical analysis

The Kaplan-Meier method was used to estimate survival time for training group and validating group. Then the two-sided log rank test was performed to compare the differences in survival times between the low-risk and high-risk groups in both sets. Furthermore, multivariate Cox analysis and data stratification analysis were performed to test whether the risk score was independent of other clinical features, including age, gender, race, pathological grade and TNM stage, which were used as covariates. *P*-values less than 0.05 were considered statistically significant.

## References

[1] Kamat AM, Hahn NM, Efstathiou JA, Lerner SP, Malmström PU, Choi W, Guo CC, Lotan Y, Kassouf W. Bladder cancer. Lancet. 2016; 388:2796–810. 10.1016/S0140-6736(16)30512-8 27345655

[2] Redelman-Sidi G, Glickman MS, Bochner BH. The mechanism of action of BCG therapy for bladder cancer—a current perspective. Nat Rev Urol. 2014; 11:153–62. 10.1038/nrurol.2014.15 24492433

[3] Antoni S, Ferlay J, Soerjomataram I, Znaor A, Jemal A, Bray F. Bladder Cancer Incidence and Mortality: A Global Overview and Recent Trends. Eur Urol. 2017; 71:96–108. 10.1016/j.eururo.2016.06.010 27370177

[4] Zhou M, Guo M, He D, Wang X, Cui Y, Yang H, Hao D, Sun J. A potential signature of eight long non-coding RNAs predicts survival in patients with non-small cell lung cancer. J Transl Med. 2015; 13:231. 10.1186/s12967-015-0556-3 26183581 PMC4504221

[5] Cheetham SW, Gruhl F, Mattick JS, Dinger ME. Long noncoding RNAs and the genetics of cancer. Br J Cancer. 2013; 108:2419–25. 10.1038/bjc.2013.233 23660942 PMC3694235

[6] Liu N, Liu Q, Yang X, Zhang F, Li X, Ma Y, Guan F, Zhao X, Li Z, Zhang L, Ye X. Hepatitis B Virus-Upregulated LNC-HUR1 Promotes Cell Proliferation and Tumorigenesis by Blocking p53 Activity. Hepatology. 2018; 68:2130–2144. 10.1002/hep.30098 29790592

[7] Lopez-Pajares V, Qu K, Zhang J, Webster DE, Barajas BC, Siprashvili Z, Zarnegar BJ, Boxer LD, Rios EJ, Tao S, Kretz M, Khavari PA. A LncRNA-MAF:MAFB transcription factor network regulates epidermal differentiation. Dev Cell. 2015; 32:693–706. 10.1016/j.devcel.2015.01.028 25805135 PMC4456036

[8] Gonzalez I, Munita R, Agirre E, Dittmer TA, Gysling K, Misteli T, Luco RF. A lncRNA regulates alternative splicing via establishment of a splicing-specific chromatin signature. Nat Struct Mol Biol. 2015; 22:370–76. 10.1038/nsmb.3005 25849144 PMC6322542

[9] Zheng P, Li H, Xu P, Wang X, Shi Z, Han Q, Li Z. High lncRNA HULC expression is associated with poor prognosis and promotes tumor progression by regulating epithelial-mesenchymal transition in prostate cancer. Arch Med Sci. 2018; 14:679–86. 10.5114/aoms.2017.69147 29765457 PMC5949918

[10] Li B, Mao R, Liu C, Zhang W, Tang Y, Guo Z. LncRNA FAL1 promotes cell proliferation and migration by acting as a CeRNA of miR-1236 in hepatocellular carcinoma cells. Life Sci. 2018; 197:122–29. 10.1016/j.lfs.2018.02.006 29421439

[11] Ye JJ, Cheng YL, Deng JJ, Tao WP, Wu L. LncRNA LINC00460 promotes tumor growth of human lung adenocarcinoma by targeting miR-302c-5p/FOXA1 axis. Gene. 2019; 685:76–84. 10.1016/j.gene.2018.10.058 30359741

[12] Zhu X, Li Y, Zhao S, Zhao S. LSINCT5 activates Wnt/β-catenin signaling by interacting with NCYM to promote bladder cancer progression. Biochem Biophys Res Commun. 2018; 502:299–306. 10.1016/j.bbrc.2018.05.076 29772237

[13] Yang K, Hou Y, Li A, Li Z, Wang W, Xie H, Rong Z, Lou G, Li K. Identification of a six-lncRNA signature associated with recurrence of ovarian cancer. Sci Rep. 2017; 7:752. 10.1038/s41598-017-00763-y 28389671 PMC5429632

[14] Song P, Jiang B, Liu Z, Ding J, Liu S, Guan W. A three-lncRNA expression signature associated with the prognosis of gastric cancer patients. Cancer Med. 2017; 6:1154–64. 10.1002/cam4.1047 28444881 PMC5463065

[15] Zhang H, Cai Y, Zheng L, Zhang Z, Lin X, Jiang N. LncRNA BISPR promotes the progression of thyroid papillary carcinoma by regulating miR-21-5p. Int J Immunopathol Pharmacol. 2018; 32:2058738418772652. 10.1177/2058738418772652 29856242 PMC5985546

[16] Shi X, Zhao Y, He R, Zhou M, Pan S, Yu S, Xie Y, Li X, Wang M, Guo X, Qin R. Three-lncRNA signature is a potential prognostic biomarker for pancreatic adenocarcinoma. Oncotarget. 2018; 9:24248–59. 10.18632/oncotarget.24443 29849937 PMC5966255

[17] Li J, Chen Z, Tian L, Zhou C, He MY, Gao Y, Wang S, Zhou F, Shi S, Feng X, Sun N, Liu Z, Skogerboe G, et al. LncRNA profile study reveals a three-lncRNA signature associated with the survival of patients with oesophageal squamous cell carcinoma. Gut. 2014; 63:1700–10. 10.1136/gutjnl-2013-305806 24522499 PMC4215280

[18] Tu Z, He D, Deng X, Xiong M, Huang X, Li X, Hao L, Ding Q, Zhang Q. An eight-long non-coding RNA signature as a candidate prognostic biomarker for lung cancer. Oncol Rep. 2016; 36:215–22. 10.3892/or.2016.4817 27222340

[19] Meng J, Li P, Zhang Q, Yang Z, Fu S. A four-long non-coding RNA signature in predicting breast cancer survival. J Exp Clin Cancer Res. 2014; 33:84. 10.1186/s13046-014-0084-7 25288503 PMC4198622

[20] Wang YL, Shao J, Wu X, Li T, Xu M, Shi D. A long non-coding RNA signature for predicting survival in patients with colorectal cancer. Oncotarget. 2017; 9:21687–95. 10.18632/oncotarget.23431 29774095 PMC5955136

[21] Zhu X, Tian X, Yu C, Shen C, Yan T, Hong J, Wang Z, Fang JY, Chen H. A long non-coding RNA signature to improve prognosis prediction of gastric cancer. Mol Cancer. 2016; 15:60. 10.1186/s12943-016-0544-0 27647437 PMC5029104

[22] Lucca I, Fajkovic H, Klatte T. Sex steroids and gender differences in nonmuscle invasive bladder cancer. Curr Opin Urol. 2014; 24:500–05. 10.1097/MOU.0000000000000092 24978392

[23] DeDeugd C, Miyake M, Palacios DA, Rosser CJ. The Influence of Race on Overall Survival in Patients with Newly Diagnosed Bladder Cancer. J Racial Ethn Health Disparities. 2015; 2:124–31. 10.1007/s40615-014-0055-x 26863249

[24] Schinkel JK, Shao S, Zahm SH, McGlynn KA, Shriver CD, Zhu K. Overall and recurrence-free survival among black and white bladder cancer patients in an equal-access health system. Cancer Epidemiol. 2016; 42:154–58. 10.1016/j.canep.2016.04.012 27161431 PMC5727912

[25] Seitz AK, Christensen LL, Christensen E, Faarkrog K, Ostenfeld MS, Hedegaard J, Nordentoft I, Nielsen MM, Palmfeldt J, Thomson M, Jensen MT, Nawroth R, Maurer T, et al. Profiling of long non-coding RNAs identifies LINC00958 and LINC01296 as candidate oncogenes in bladder cancer. Sci Rep. 2017; 7:395. 10.1038/s41598-017-00327-0 28341852 PMC5428251

[26] Yazarlou F, Modarressi MH, Mowla SJ, Oskooei VK, Motevaseli E, Tooli LF, Nekoohesh L, Eghbali M, Ghafouri-Fard S, Afsharpad M. Urinary exosomal expression of long non-coding RNAs as diagnostic marker in bladder cancer. Cancer Manag Res. 2018; 10:6357–65. 10.2147/CMAR.S186108 30568497 PMC6267766

[27] Guo E, Liang C, He X, Song G, Liu H, Lv Z, Guan J, Yang D, Zheng J. Long Noncoding RNA LINC00958 Accelerates Gliomagenesis Through Regulating miR-203/CDK2. DNA Cell Biol. 2018; 37:465–72. 10.1089/dna.2018.4163 29570358

[28] Langfelder P, Horvath S. WGCNA: an R package for weighted correlation network analysis. BMC Bioinformatics. 2008; 9:559. 10.1186/1471-2105-9-559 19114008 PMC2631488

